# Definitive carbon ion radiotherapy for tracheobronchial adenoid cystic carcinoma: a preliminary report

**DOI:** 10.1186/s12885-021-08493-1

**Published:** 2021-06-26

**Authors:** Jian Chen, Jingfang Mao, Ningyi Ma, Kai-Liang Wu, Jiade Lu, Guo-Liang Jiang

**Affiliations:** 1 Department of Radiation Oncology, Shanghai Proton and Heavy Ion Center, Shanghai, 201321 China; 2 Shanghai Key Laboratory of Radiation Oncology, Shanghai Engineering Research Center of Proton and Heavy Ion Radiation Therapy, Shanghai, 201321 China; 3 Department of Radiation Oncology, Shanghai Proton and Heavy Ion Center, Fudan University Cancer Hospital, Shanghai, 201321 China

**Keywords:** Tracheobronchial carcinoma, Adenoid cystic carcinoma, Carbon ion radiotherapy

## Abstract

**Background:**

Tracheobronchial adenoid cystic carcinoma (TACC) is a rare tumour. About one-third of patients miss their chance of surgery or complete resection as it is mostly detected in the advanced stage; hence, photon radiotherapy (RT) is used. However, the outcomes of photon RT remain unsatisfactory. Carbon ion radiotherapy (CIRT) is thought to improve the therapeutic gain ratio; however, the outcomes of CIRT in TACC are unclear. Therefore, we aimed to assess the effects and toxicities of CIRT in patients with TACC.

**Methods:**

The inclusion criteria were as follows: 1) age 18–80 years; 2) Eastern Cooperative Oncology Group Performance Status 0–2; 3) histologically confirmed TACC; 4) stage III–IV disease; 5) visible primary tumour; and 6) no previous RT history. The planned prescription doses of CIRT were 66–72.6 GyE/22–23 fractions. The rates of overall survival (OS), local control (LC), and progression-free survival (PFS) were calculated using the Kaplan-Meier method. Treatment-induced toxicities and tumour response were scored according to the Common Terminology Criteria for Adverse Events and Response Evaluation Criteria in Solid Tumors, respectively.

**Results:**

Eighteen patients with a median age of 48 (range 30–73) years were enrolled. The median follow-up time was 20.7 (range 5.8–44.1) months. The overall response rate was 88.2%. Five patients developed lung metastasis after 12.2–41.0 months and one of them experienced local recurrence at 31.9 months after CIRT. The rates of 2-year OS, LC, and PFS were 100, 100, and 61.4%, respectively. Except for one patient who experienced grade 4 tracheal stenosis, which was relieved after stent implantation, no other ≥3 grade toxicities were observed.

**Conclusions:**

CIRT might be safe and effective in the management of TACC based on a short observation period. Further studies with more cases and longer observation are warranted.

## Background

Salivary gland tumours are rare diseases that could affect head, neck, breast, pelvis, gynaecological tract, and trachea [1–6]. Twenty-two percent of malignant salivary gland tumours are adenoid cystic carcinomas (ACCs). Tracheobronchial adenoid cystic carcinoma (TACC) originates from the submucosal glands of the tracheobronchial tree, and accounts for only 10% of tracheal tumours. The incidence of primary tracheal tumours is < 0.2 per 100,000 persons per year in the United States [7–10]. TACC grows slowly, is mostly asymptomatic in the early stage, and is often discovered at an advanced stage [11]. Surgical resection plays an important role in treatment. However, by the time of diagnosis, about one-third of patients have missed their chance of surgery or complete resection due to the limited resection scope of the trachea and the tumour’s characteristic spread along the bronchial wall and/or nerves at an early stage [12]. It has been reported that a positive surgical margin (R1 or R2 resection) after surgery could occur in 50% [13]–84.4% [14] of all cases.

X-ray radiation therapy (RT), as an adjuvant or definitive treatment method, has been used for TACC. In the early years, the clinical outcomes of definitive RT for TACC were not satisfactory as it is a slowly-growing malignancy and resistant to RT [9, 15–17]. Modern RT techniques such as intensity-modulated radiation therapy (IMRT) have provided improved results [13, 18]. As TACC is a very rare disease, no prospective study on it has been conducted. Only a few retrospective studies have been published to date, and less than 300 TACC patients receiving definitive RT have been reported [11–13, 15–25]. Therefore, the exact role of RT remains unclear.

Charged particle beams, including proton and carbon ion beams, have physical advantages, such as specific dose distribution of the Bragg peak and narrow penumbra. Therefore, they could provide better sparing of normal tissue [26–28]. In addition, carbon ions are characterized by high linear energy transfer (LET), and thus, carbon ion beam produces a stronger biological effect in killing tumour cells with high relative biological effect (RBE), especially for radio-resistant tumours [29, 30]. The synergy of these two features provides a critical advantage in radio-resistant malignancies. In head and neck ACC, Jensen et al. [31] found that photon plus carbon ion radiotherapy (CIRT) boost could improve local control (LC) and survival when compared to photon therapy only. Högerle et al. [13] reported on 38 TACC patients treated with surgery and/or radiotherapy with either CIRT or photons. The 5-year overall survival (OS), freedom from local progression, and freedom from distant progression in patients who underwent RT alone and multimodal treatment including surgery and adjuvant RT were 100 and 84%, 88 and 100%, and 67 and 65%, respectively. Two patients received CIRT only, one for adjuvant therapy and another for definitive therapy. A high LC rate was achieved 20 months after CIRT.

Our centre, Shanghai Proton and Heavy Ion Center (SPHIC) was officially opened in 2015. Since then, we have treated 23 patients having TACC with CIRT. Here, we retrospectively summarize the preliminary results.

## Methods

### Patients and pretreatment evaluations

Patients who met the following criteria were enrolled onto in this study: 1) 18–80 years old; 2) Eastern Cooperative Oncology Group Performance Status 0–2; 3) histologically confirmed TACC; 4) stage III–IV disease according to the modified Bhattacharyya’s protocol [13]; 5) visible primary tumour; and 6) no previous RT history. The study was approved by the IRB of SPHIC (approval number SPHIC-TR-2017-02, RS). Informed consent was obtained from all the participants.

Pretreatment evaluation in all patients included complete disease history and physical examination, complete blood count, serum electrolytes, renal and liver function tests, electrocardiogram, pulmonary function tests, and a mandatory fluorodeoxyglucose positron emission tomography/computed tomography (FDG-PET/CT) scan for clinical staging, same as pretreatment evaluation for lung cancer patients we reported before [32]. Bronchoscopy was required for all patients. For patients whose esophagus was suspiciously invaded by tumour, ultrasound esophagoscopy/gastroscopy was mandatory before CIRT.

### Preparing, planning and delivery of CIRT

The procedures of CIRT, including preparing, planning and delivery, was performed similar to previously described [32]. The patients were immobilized in the supine position using thermoplastic masks with either an AlphaCradle® (for lesions located in the upper part of the trachea) or a vacuum bag (for lesions located in the lower part of the trachea or bronchus) to immobilize the patient’s body position and restrict the breath motion for patients using free breathing (FB) or gating.

The scanning scope of the simulation computer tomography (CT) starts from the angle of the mandible to the adrenal glands to include tumour lesions, entire lungs, whole neck, and all the organs/tissues through which the beams were likely to pass. All patients were evaluated for tumour motion using 4-dimensional (4-D) simulation CT. If the tumour motion in any direction was less than 5 mm, the patient was treated under FB; if the motion exceeded 5 mm, a breath control technique, either active breathing control (ABC, Elekta Oncology Systems, Crawley, UK) or respiratory gating (Anzai Respiratory Gating System, AZ-733 V, Anzai Medical Co. Ltd., Japan), was required to mitigate the residual motion (RM) to < 5 mm during treatment. For patients using gating, 10 phases of the whole respiratory cycle were reconstructed on 4-D CT. The gating window (respiratory phase time when the beam is on, usually around the end of exhalation) was selected to restrict the RM to < 5 mm, same as breath control methods for lung cancer patients we reported before [32].

The target delineation was also similar as our protocol for lung cancer patients [32]. Gross tumour volume (GTV) was determined according to contrast thoracic CT and PET/CT or magnetic resonance imaging (MRI). For patients using the gating technique, an internal gross tumour volume (iGTV) was created by combining the GTVs of all respiratory phases. The clinical target volume (CTV) was defined as a 0.5–1.0 cm expansion in the circumferential direction and a 1.0–2.0 cm (2.0 cm in 15 of 18 patients) margin in the longitudinal direction. Range uncertainties and set-up errors were taken into account when creating the planning target volume (PTV). In most instances, it was CTV plus a 0.3–0.5 cm lateral margin and a 0.5–1.2 cm margin along the beam direction. The dose of CIRT was defined as the equivalent dose to Gy of photon (GyE). The relative biological effective dose was calculated based on the local effect model I, with typical RBEs within the spread out bragg peak (SOBPs) of about 3.0 ~ 5.0 [33]. The prescription doses were 66–72.6 GyE in 22–23 fractions, 5 fractions a week. A metastatic lesion in one patient’s right lower lobe detected before CIRT was treated using stereotactic body radiotherapy (SBRT) with a total dose of 60 Gy in 10 fractions, 5 fractions a week.

The target coverage requirements were as follows: (i) at least 99% of the GTV was covered by 99% of the prescription dose, (ii) 99% of the CTV by 95% of the prescription dose, and (iii) 90% of the PTV by 90% of the prescription dose. The dose constraints for the main organs at risk (OARs) included: (1) lung: mean dose (Dmean) of bilateral lungs < 15 GyE, the percentage volume of the lung receiving 20 GyE or more in total lung (V20) < 20%, V5 < 50%; maximum dose (Dmax) of the main bronchial tree < 105% of the prescription dose; (2) heart: V30 < 30%, V40 < 25%; (3) oesophagus: Dmean < 34 GyE, Dmax < 105% of the prescription dose; (4) spinal cord: Dmax < 45 GyE; (5) thyroid: Dmean < 45 GyE; and (6) stomach: Dmax < 45 GyE.

The Siemens Syngo Planning System® was used for planning in all patients. Beam energy 85–430 MeV (CIRT) plans were designed using 2–4 beams with the PBS technique. Two orthogonal X-ray images were taken to verify the patient’s position according to bone structures before each daily irradiation. A set-up error ≤ 3 mm before the treatment was allowed. Carbon ion beams were delivered under the same breath control mode used for simulation CT. All the patients underwent review CT before the first treatment and every week during treatment, and plan recalculation on the latest CT was conducted for every patient to check the dose distribution. Replanning was demanded when recalculation revealed poor coverage of targets or overdosing to the organs at risk. These procedures were same as treatment protocols for lung cancer patients we treated before [32].

### Follow-up and evaluation

This part was similar to previously described [32]. All patients were evaluated weekly for treatment-induced toxicities and disease response/progression during treatment. If the patient had any symptoms or signs of airway stenosis or disease recurrence, bronchoscopy was strongly recommended. After the completion of CIRT, all patients were required to be evaluated 3 months after the 1st day of CIRT, every 3–4 months within the first 2 years, every 6 months between years 3 and 5, and annually thereafter. Treatment-induced side effects were scored according to the Common Terminology Criteria for Adverse Events, version 4.03. Toxicities occurring 90 or more days after the initiation of CIRT were defined as late toxicities. The Response Evaluation Criteria in Solid Tumors, version 1.1, was used for tumour response evaluation.

### Statistical analyses

The objective response rate (ORR) was defined as the proportion of patients who reached complete response (CR) or partial response (PR) and kept for more than 3 months in all patients. The rates of overall survival (OS), local control (LC), and progression free survival (PFS) were calculated using the Kaplan-Meier method. The times to events were calculated from the start of CIRT until the first documented treatment failure. OS was defined till the date of death or the last follow-up. LC was defined till the date of local failure or the last follow-up. PFS was defined till the date of disease progression at any site or death, or the last follow up. All analyses were performed using SPSS® statistics version 26 (Armonk, NY, USA).

## Results

### Patient characteristics

From March 2016 to December 2019, 23 consecutive patients with TACC received CIRT in the SPHIC. Among them, three patients previously received RT; two treated by radical surgery with positive surgical margins but had no visible residual tumour (R1 resection). Hence, 18 patients who met the inclusion criteria were enrolled in this analysis. Five patients were treatment-naive, two underwent R2 resection, one underwent exploratory surgery, three had recurrent tumour 1.2–30 years after surgery, and seven underwent endoscopic debulking surgery (including argon helium laser ablation, cryosurgical ablation, and endoscopic trepanned resection) before CIRT. One patient had one lung metastatic lesion and one had multiple lung metastases before CIRT. The lesions in six patients involved the carina or bilateral main bronchus. Thirteen patients had lesions that were longer than 5 cm. Patient characteristics are detailed in Table 1.

**Table 1:** Characteristics of patients and treatment

Characteristic	Value
No. of patients	18
Age at treatment, y	
Median	48.0
Range	30–73
Sex	
Male	10
Female	8
ECOG score	
0	14
1	4
Smoking history	
Yes	5
No	13
Diameter on transversal section, mm	
Median	34.0
Range	19.0–54.0
Length on craniocaudal direction, mm	
Median	61.5
Range	27.0–109.0
GTV Volume, cm^3^	
Median	42.675
Range	5.61–87.51
Stage^a^:	
III	7
T3N0M0	7
IV	11
T1N1M0	1
T3N1M0	3
T4N0M0	5
T4N0M1^b^	2
Chemotherapy	
Yes	2
No	16
Carbon ion radiotherapy	
66 GyE / 22 fractions	1
69 GyE / 23 fractions	10
72.6 GyE / 22 fractions	5
82.5–85.8 GyE / 25–26 fractions	2

*ECOG* Eastern Cooperative Oncology Group, *GTV* gross tumor volume

^a^Modified Bhattacharyya staging system [13].

^b^One patient had one solid lung metastatic lesion, and one patient had multiple lung metastases before carbon ion radiotherapy

### CIRT

Among 18 patients, the methods used for breathing control were ABC in 1 patient, respiratory gating in 9 patients, and FB in 8 patients.

Sixteen patients received a total dose of 66–72.6 GyE/22–23 fractions (Table 1). The two patients who received more than 72.6 GyE experienced treatment interruptions of 25 days and 2 months due to infectious pneumonia and whole lung atelectasis by mucosal oedema in the extremely narrowed bronchus after surgery, respectively. The compensated dose was decided by the physician’s discretion after balancing tumour cell re-proliferation and the protection of OARs.

### Toxicities

All patients tolerated CIRT well. No patient experienced grade 3 or higher acute toxicities. Grade 2 acute toxicities included oesophagitis (two cases), pneumonitis (one), tracheal stenosis (one), hoarseness (one), and haematological toxicities (one). Regarding late toxicities, airway stenosis was observed in three patients. Among them, one patient who underwent argon helium laser ablation plus cryosurgical ablation before CIRT experienced grade 4 tracheal stenosis 4.5 months from the first fraction of CIRT, and the stenosis was relieved after stent insertion. The second patient received similar endoscopic debulking treatment before RT and developed grade 2 tracheal stenosis 1.2 months from the first fraction of CIRT, and symptoms improved after symptomatic treatment. The third patient, whose lesion was located close to the glottis, experienced grade 2 laryngostenosis 8 months after the first fraction of CIRT, and the symptoms were relieved after symptomatic therapy. The median maximal doses to the trachea/larynx of patients with or without airway stenosis were 72.91 GyE (range 68.69 ~ 73.19) versus 72.66 GyE (range: 69.73 ~ 87.41). Other grade 2 late toxicities included radiation-induced lung injury (RILI) (three cases), hypothyroidism (one), and tracheitis (one) (Tables 2 and 3).

**Table 2:** Acute toxicities of the entire cohort

Acute toxicity	Grade (percentage, %)		
	1	2	3 ~ 5
Esophagitis	12 (66.7)	2 (11.1)	0 (0.0)
Pneumonitis	3 (16.7)	1 (5.6)	0 (0.0)
Neutropenia	3 (16.7)	1 (5.6)	0 (0.0)
Hoarseness	2 (11.1)	1 (5.6)	0 (0.0)
Tracheal stenosis	0 (0.0)	1 (5.6)	0 (0.0)
Dermatitis	6 (33.3)	0 (0.0)	0 (0.0)
Cough	6 (33.3)	0 (0.0)	0 (0.0)
Leukocytopenia	6 (33.3)	0 (0.0)	0 (0.0)
Anemia	1 (5.6)	0 (0.0)	0 (0.0)

**Table 3:** Late toxicities of the entire cohort

Late toxicity		Grade (percentage, %)			
	1	2	3	4	5
Tracheal stenosis	0 (0.0)	1 (5.6)	0 (0.0)	1 (5.6)	0 (0.0)
RILI	5 (27.8)	3 (16.7)	0 (0.0)	0 (0.0)	0 (0.0)
Tracheitis	0 (0.0)	1 (5.6)	0 (0.0)	0 (0.0)	0 (0.0)
Laryngostenosis	0 (0.0)	1 (5.6)	0 (0.0)	0 (0.0)	0 (0.0)
Hypothyroidism	0 (0.0)	1 (5.6)	0 (0.0)	0 (0.0)	0 (0.0)
Pleural effusion	2 (11.1)	0 (0.0)	0 (0.0)	0 (0.0)	0 (0.0)
Skin	1 (5.6)	0 (0.0)	0 (0.0)	0 (0.0)	0 (0.0)

*RILI* radiation-induced lung injury

### Clinical outcomes

**Fig. 1:**
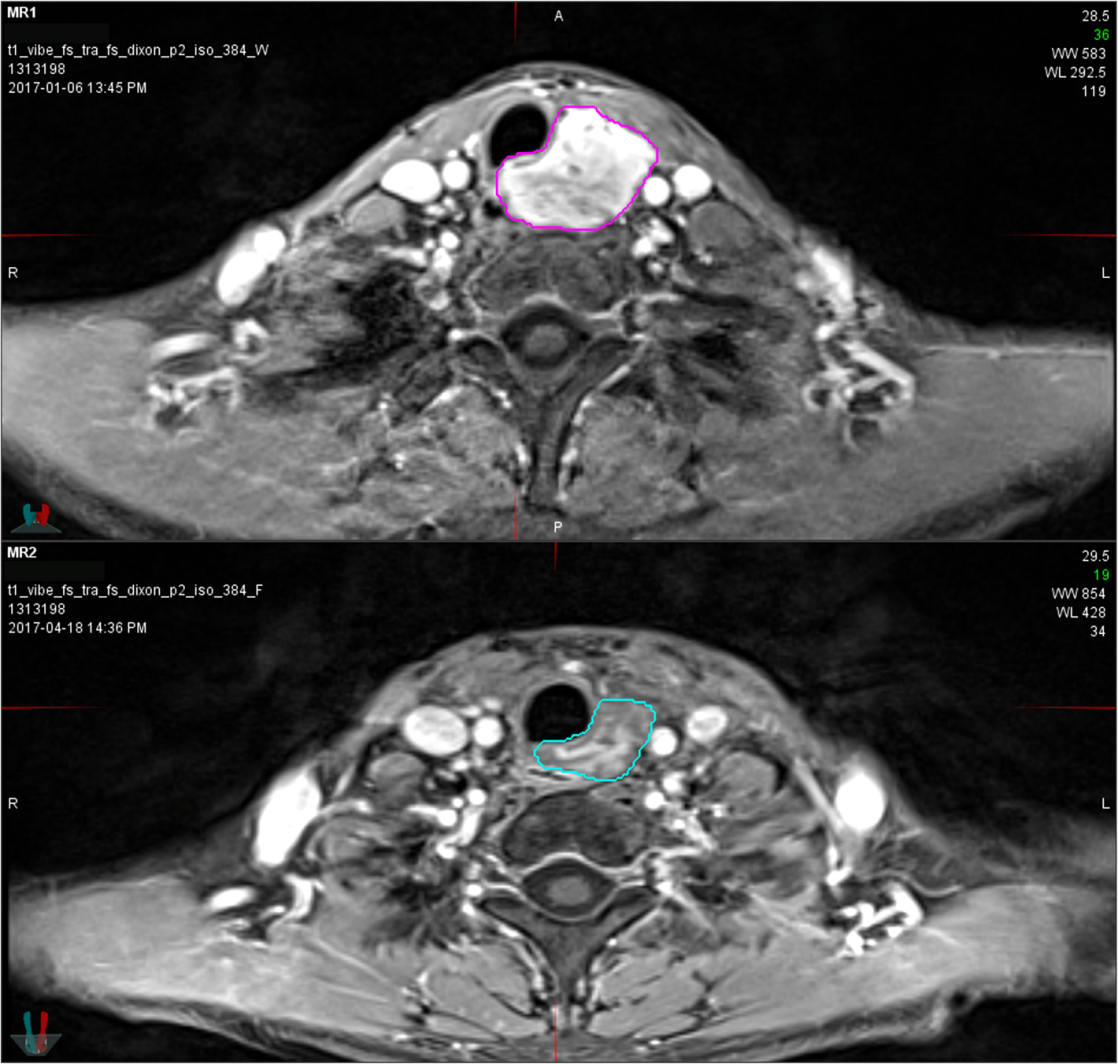
A typical case. A typical case of tracheal adenoid cystic carcinoma located in the upper third portion of the trachea. The patient was treated with carbon ion radiotherapy of 69 GyE in 23 fractions. The tumour shrank significantly 3 months after carbon ion radiotherapy and was evaluated as partial response

**Fig. 2:**
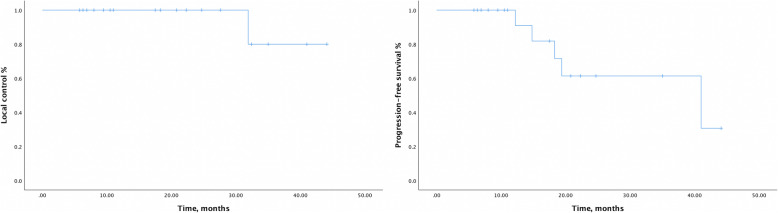
Local control and progression-free survival. Local control (LC) and progression-free survival (PFS) after carbon ion radiotherapy in 18 patients

On June 30, 2020, the median follow-up time was 20.7 (range 5.8–44.1) months. Tumour response at 3 months after CIRT was evaluated in 17 patients, except for 1 patient, whose tumour was not shown clearly on CT due to the atelectasis. ORR was 88.2% with CR in 7, PR in 8, and stable disease (SD) in 2 patients. Five distant metastases occurred in five patients during follow-up. Among them, one patient with stage IV (T4N0M0) disease developed multiple lung metastases after 14.8 months and experienced local recurrence 31.9 months from the first fraction of CIRT with a dose of 69 GyE/23 fractions. One or more newly emerged metastatic lung lesions were observed in the other four patients 12.2–41.0 months from the first fraction of CIRT. For salvage treatments, one patient received radioactive seed implantation for local recurrence, and one SBRT for lung metastases. The median PFS time in all patients was 41.0 (95% confidence interval [CI], 9.3–72.7) months. The rates of 2-year OS, LC, and PFS were 100, 100, and 61.4%, respectively, for the whole cohort. A typical case is shown in Fig. 1. The 2-year LC and PFS are shown in Fig. 2.

## Discussion

TACC is a very rare tumour, and the clinical experience is limited. Although surgery plays the most critical role, radical resection is often difficult. RT has not been recognized for its role in the management of TACC. Published data on radical or definitive RT for TACC patients have been limited to small sample sizes. Compared to surgery with or without adjuvant RT, definitive RT usually resulted in inferior clinical outcomes in several retrospective analyses (see Table 4). In reports published before 2012, the 5-year OS rates varied from 40 to 53% [9, 15–17]. More recently, definitive RT was reported to have improved outcomes in some studies with small sample sizes, with a 5-year OS of 63.7–86% [11, 18, 24]. The 5-year LC rates reached 90–100% as reported by Je et al. and Levy et al. in only 9 patients each [11, 18].

**Table 4:** Outcomes of surgery with/without postoperative radiotherapy and radiotherapy only for tracheobronchial adenoid cystic carcinoma

Author	Year	Case number	Therapy	MST (month)	5-y LC (%)	5-y OS (%)	10-y LC (%)	10-y OS (%)
**Grill** **[**19**]**	1990	45	S + R	118				
		12	R	28				
**Maziak** **[**20**]**	1996	36	S +/− R	87				
		6	R	73				
**Kanematsu** **[**16**]**	2002	11	S +/− R			91		76
		5	R			40		0
**Molina** **[**9**]**	2007	24	S +/− R			70		63
		16	R			53		31
**Lee** **[**17**]**	2011	17	S +/− R			100		90
		13	R +/− C			54		27
**Shadmehr** **[**15**]**	2011	13	S + R	68.8		78		
		5	R	21.2		40 (2-y)		
**Je** **[**18**]**	2017	13	S + R		100	92.3	100	76.9
		9	R		100	66.7	26.7	22.2
**Levy** **[**11**]**	2018	22	S + R		100	82		
		9	R		90	86		
**Högerle** **[**13**]**	2019	7	S		100	100	100	80
		13	S + R		100	92	100	82
		18	R		86	100	43	83
**Wang** **[**24**]**	2019	156	S +/− R	198		85		63.4
		27	R	92		63.7		46.4

*MST* median survival time, *LC* local control, *OS* overall survival, *S* surgery, *R* radiotherapy, *C* chemotherapy

Regarding the application of particle RT (neutron, proton, and heavy ion beams) in TACC, a few studies have been published. Bittner et al. [22] reported 2-year LC and OS rates of 89 and 89.4%, respectively, in 20 patients, when using neutrons. There were two cases of grade 3/4 chronic toxicities, including one case of tracheal stenosis and one of brachial plexopathy. Neutron RT has been abandoned globally in RT society because of severe toxicity. Verma et al. [25] reported results of proton RT in five TACC patients. With a median follow-up of 10 months, no recurrent disease was observed. One patient developed bronchial stenosis and required a stent. The study conducted by Högerle et al. was the only one, which used CIRT to treat TACC; however, only two patients were treated with CIRT alone, and another five were treated with combined photon and carbon ion therapy [13]. It is therefore, difficult to determine the efficacy and toxicities of CIRT in TACC patients with such a small sample.

TACC is thought to be a slowly growing malignancy and with radio-resistance. CIRT has been demonstrated to have biological advantages on radio-resistant malignancies in in-vitro and in-vivo experiments, as well as in clinical practice [34]. Its capability to sterilize malignancies is 2–3 times stronger than that of a conventional X-ray RT with RBE of 2–3, especially for radio-resistant tumours [29]. Apart from the biological advantage, CIRT also has advantages such as dose distributions of Bragg peak, which could spare normal structures around the targets significantly [26]. In the current study, we have successfully delivered CIRT of 69–72.6 GyE in 15 out of all 18 TACC patients. Overall, the acute and late toxicities were mild. Only one patient experienced grade 4 tracheal stenosis. The maximal doses to the trachea/larynx were similar between patients with and without airway stenosis of any grade. Fisher’s exact test showed the rate of stenosis occurrence was not different between patients received intra-tracheal ablation and those did not (*p* = 0.528). Whether the stenosis was caused by the intra-tracheal laser ablation, the nature of an intraluminal malignancy, or individual radiation sensitivity of trachea, etc. remains unclear and need to be further investigated. No other grade ≥ 3 adverse effects were observed. The 2-year LC and OS rates for the whole cohort reached 100% with a median follow-up time of 20.7 months. Only one patient developed local recurrence at 31.9 months from the first fraction of CIRT and, including this patient, five had distant metastatic (DM) lesions to the lungs. Our results using CIRT are promising compared to those using photon or neutron beams.

There are currently no up to date guidelines on the CIRT technique that we could follow. TACC tends to have mucosal or sub-mucosal spread along the tracheal wall; the subclinical lesion is at least 1 cm beyond the visible or palpable tumours [35]. To cover the subclinical lesion, some authors used a 3-cm margin beyond the GTV to create the CTV [18, 24]. In this study, the CTV was defined in most cases as the GTV plus margins of 2 cm longitudinally and 0.5–1 cm circumferentially. At the last follow-up, no marginal recurrences were observed. Additionally, as the regional lymph node involvement rate was low, and given that lymph node involvement has little impact on prognosis, [19] prophylactic lymph node drainage area irradiation was not recommended. In this study, elective nodal irradiation was not applied, and no regional lymph node recurrence was observed. These results suggest the suitability of the radiation field design of this study; and the same strategy has been implemented in our phase II prospective study for TACC. Without guidelines, we were also unsure of the exact dose and fractionation to be delivered. From the literature, doses as high as 56–60 Gy were needed for X-ray RT [21, 36]. In some studies, brachytherapy was used to increase the dose to as high as 74.4 Gy, and the 5-year LC rate reached 100% [13, 18]. However, endobronchial brachytherapy was associated with higher fatal haemoptysis, ulcers, necrosis, or stenosis of the bronchi as late severe respiratory complications. Furthermore, brachytherapy was not suitable in patients with extrabronchial spread or regional lymph node metastasis [37–39]. In this study, 66–72.6 GyE/22–23 Fx of carbon ion doses were used and a 100% 2-year LC was achieved with mild toxicities. The dose seemed effective and well tolerated, and CIRT could provide higher tumour control probability (TCP) without significantly increasing the normal tissue complication probability (NTCP).

In this study, the most prominent late toxicity was airway stenosis, which can be life threatening. Airway stenosis was observed in other published reports, using either surgery or radiation therapy, and the incidence varied from 5 to 23% [11, 18, 22, 25]. In our study, 3 of 18 patients (17%) developed tracheal stenosis with Grade 4 in 1 and Grade 2 in 2. Two of 3 received intra-tracheal surgery or laser ablation before CIRT, which most likely attributed to the stenosis, and the third one had a lesion in his glottis. One patient developed entire right lung atelectasis during CIRT, and the cause of it was likely the prior surgery, which involved the right main bronchi. As TACC originates from the tracheal epithelium, it is easy to develop airway stenosis, especially in patients undergoing prior surgery or ablation. Therefore, we should always be aware of this risk, and adopt necessary preventive measurements.

Lung metastasis was an important outcome in the patients in this study. Five developed new metastases to the lungs after CIRT; the 2-year DM rate was 38.6%. This is consistent with previous reports that the 5-year lung metastasis rate could be as high as 77.8% (20–77.8%) [11, 13, 18]. The development of systemic treatment seems to be important. However, chemotherapy provided very limited benefit for ACC patients with lung metastases, [40] and an effective target therapy is still under development. A phase II study showed that lenvatinib had an ORR of 15.6% for recurrent TACC, while 18 of 32 patients discontinued therapy because of drug-related issues [41]. Therefore, it is a great challenge to develop new medications to further improve TACC outcomes.

There were some limitations to this study. First, the number of patients was small. Second, the compensated dose for interruptions of CIRT was decided by the physician’s discretion as there was no established compensation method and dose constraints to OARs for CIRT to follow. Third, the follow-up time was not long enough, especially for this slowly growing tumour, and thus, local control and survival rates may have not reached their final values. Prospective studies with large sample sizes are warranted to better define the role of CIRT in patients with TACC. Two prospective phase II clinical trials involving patients with different surgical statuses are ongoing in our centre.

## Conclusions

To the best of our knowledge, this is the first report on TACC treated only by CIRT for all the patients. This study showed that CIRT was feasible; toxicities were mild, and overall survival and local control at 2-years were satisfactory. However, more patients and long-term follow-up are needed to confirm the findings shown in this study.

## Data Availability

The datasets used in the current study available from the corresponding author on reasonable request.
